# Detecting the Norovirus Season in Sweden Using Search Engine Data – Meeting the Needs of Hospital Infection Control Teams

**DOI:** 10.1371/journal.pone.0100309

**Published:** 2014-06-23

**Authors:** Michael Edelstein, Anders Wallensten, Inga Zetterqvist, Anette Hulth

**Affiliations:** 1 Department of Epidemiology and Evaluation, Public Health Agency of Sweden, Solna, Sweden; 2 European Program for Investigation Epidemiology Training (EPIET), European Centre for Disease Prevention and Control (ECDC), Stockholm, Sweden; Inserm & Universite Pierre et Marie Curie, France

## Abstract

Norovirus outbreaks severely disrupt healthcare systems. We evaluated whether Websök, an internet-based surveillance system using search engine data, improved norovirus surveillance and response in Sweden. We compared Websök users' characteristics with the general population, cross-correlated weekly Websök searches with laboratory notifications between 2006 and 2013, compared the time Websök and laboratory data crossed the epidemic threshold and surveyed infection control teams about their perception and use of Websök. Users of Websök were not representative of the general population. Websök correlated with laboratory data (b = 0.88-0.89) and gave an earlier signal to the onset of the norovirus season compared with laboratory-based surveillance. 17/21 (81%) infection control teams answered the survey, of which 11 (65%) believed Websök could help with infection control plans. Websök is a low-resource, easily replicable system that detects the norovirus season as reliably as laboratory data, but earlier. Using Websök in routine surveillance can help infection control teams prepare for the yearly norovirus season.

## Introduction

Norovirus is a leading cause of gastroenteritis [1], responsible for sporadic cases as well as outbreaks [2]. In the United States alone, it causes between 19 and 21 million cases of acute gastroenteritis annually [1]. Norovirus outbreaks peak during winter months in temperate climates [3] and are therefore referred to as ‘winter vomiting disease’. Other pathogens, such as rotavirus, also contribute to acute gastrointestinal illness in the winter months and can lead to several peaks of gastrointestinal illness during that season [4]. In Northern Europe, winter vomiting disease occurs every year [5], [6], [7] although the onset of the season may vary [7]. Norovirus activity increases with decreasing temperature and humidity [8]. Infection with norovirus is characterized by acute onset of nausea, vomiting, abdominal cramps, myalgia, and non-bloody diarrhea. Symptoms usually resolve in 2–3 days [9]. Almost 90% of individuals with norovirus illness do not seek healthcare [10] and estimations suggest that national surveillance systems only capture 1 in 1,500 community cases [11]. During the winter vomiting disease season, community transmission of norovirus is typically followed by healthcare associated outbreaks [5]. Healthcare associated outbreaks affect patients and disrupt hospital functioning because of containment procedures (e.g., closure of wards, postponement of surgery) and staff absenteeism [2], [5], [12]. They are also costly: a 2011 study estimated that each nosocomial norovirus infection case cost $6 237 [13]. Early implementation of prevention measures including hand hygiene, staff exclusion and disinfection shorten norovirus outbreaks [14] and reduce hospital costs [13].

In Sweden, the incidence of winter vomiting disease in the community is unknown although norovirus causes 60% of all gastroenteritis outbreaks [6]. As of 2012, surveillance relied on laboratory notification, mainly reflecting hospitalized cases. Such systems do not provide information on community transmission, leading to under-ascertainment, and are subject to reporting lags [7].

Analyzing patterns of words entered in online search engines is an alternative surveillance method to obtain information on outbreaks. This technique was first piloted for influenza surveillance [15] and can be performed with syndromic and disease-specific terms [16]. However, the ability of word pattern analysis using generic search engines to accurately predict influenza outbreaks or estimate their magnitude has been questioned [17], [18], [19]. Health websites provide better epidemiological information for internet-based surveillance than generic search engines [17], [20]. Word pattern analysis based surveillance has also been tested for acute gastrointestinal illness [21] and norovirus specifically [22]. Search patterns for gastroenteritis-related terms correlated with laboratory notification patterns [21], [22]. Likewise, the volume of calls related to vomiting queries to a national health helpline provided a timely indicator of forthcoming healthcare-associated norovirus outbreaks [7]. In 2007, The Swedish institute for Communicable disease control (SMI, reorganised into Public Health Agency of Sweden since January 2014) started ‘Websök’, a system that routinely analyzes data generated by search queries entered by the public in åwww.vårdguiden.se, the official health portal for Stockholm county. Since 2009, SMI has been using Websök for influenza-like illness surveillance [23]. In 2010, a preliminary analysis of Websök use for norovirus suggested that the pattern of winter vomiting disease related searches followed the laboratory notification pattern [24] and was not influenced by mentions of winter vomiting disease in the Swedish media or by other pathogens [24]. In addition, Websök might detect the onset of the winter vomiting disease season earlier than laboratory based surveillance [24]. An early warning for the beginning of the norovirus season can help infection control teams put measures in place in a timely manner [7]. Well prepared institutions suffered less disruption, morbidity and cost [25]. Although preliminary information about Websök is encouraging, the system has not been formally evaluated. We therefore evaluated whether Websök is an accurate, timely and representative tool for the surveillance of winter vomiting disease, as a complement to laboratory surveillance, and whether its use adds public health value.

## Methods

In order to evaluate Websök's data accuracy, timeliness, representativeness and usefulness, we determined whether: (i) Websök's data correlated with voluntary laboratory reporting data (ii) Websök detected the winter vomiting season earlier than laboratory surveillance (iii) Vårdguiden.se users were representative of the Swedish population (iv) There was an added public health value to earlier detection of the winter vomiting disease season.

The query logs do not record any personal information. We could therefore not link online searches to any identifiable information, and therefore did not seek ethical committee clearance. We used STATA version 12 for all statistical analyses.

### Evaluating data accuracy

From week 27 in 2006 to week 26 in 2013, we counted the weekly number of laboratory notifications from 16 regional laboratories across Sweden, using the dates of notification to SMI, and the number of weekly searches for two norovirus related search terms: “kräk” (vomiting) and “vinterkräksjuka” (winter vomiting disease), using the dates when the searches occurred, obtained from the vårdguiden.se website logs. Searches for the term “kräk” included searches for longer words with the term “kräk” in them, such as “vinterkräksjuka”. To standardize the data, adjust for trends, and smooth, we expressed the weekly number of notifications and searches for each term as the proportion of the total number of notifications or searches for each season (from week 27 one year to week 26 the next year) and used a five week moving average. We compared Websök and laboratory surveillance data in terms of trends over time, by cross correlating smoothed laboratory notifications with smoothed weekly searches for each search term over the specified period.

### Evaluating timeliness

We defined the onset of the yearly winter vomiting season as the exceedance of the upper prediction interval of baseline norovirus activity (epidemic threshold) [26], itself defined by fitting harmonic functions on the time period with no or little activity [27]. Based on visual inspection of the data, we used the period between June through October (weeks 23 to 44, or 43 when week 44 starts in November) as baseline for laboratory data and weekly searches for “kräk” and “vinterkräksjuka”. We calculated 95% and 99% prediction intervals. For each season between 2006–07 and 2012–13, we determined the week where the number of notifications or searches exceeded the epidemic thresholds. We then measured the time interval between the epidemic thresholds crossing for the laboratory data and each Websök search term, both for individual seasons as well as the mean for the whole study period.

### Evaluating representativeness

We obtained vårdguiden.se users' characteristics from a 2012 survey of 3,000 users and the 2012 general Swedish population characteristics from the Statistics Sweden website (www.scb.se, accessed 2014 June 2). We compared vårdguiden.se users with the general population in terms of age, sex, educational attainment, and county of residence using Chi-square goodness of fit tests. Google analytics data identified the counties from which users had accessed vårdguiden.se.

### Evaluating usefulness

We prepared a questionnaire using survey generator (http://www.alstra.se/sv/enkatverktyg, accessed 2014 June 2) following in depth interviews with two infection control teams. The questionnaire contained questions regarding experience of hospital norovirus outbreaks, usual triggers for implementing norovirus control strategies, use of norovirus surveillance information, perception of internet-based surveillance data and perception of the usefulness of an early warning to the norovirus season. All county infection control teams in Sweden received the questionnaire. Non responding teams received up to two reminders. We analysed the survey results using MS Excel.

## Results

### Data accuracy

**Figure 1:**
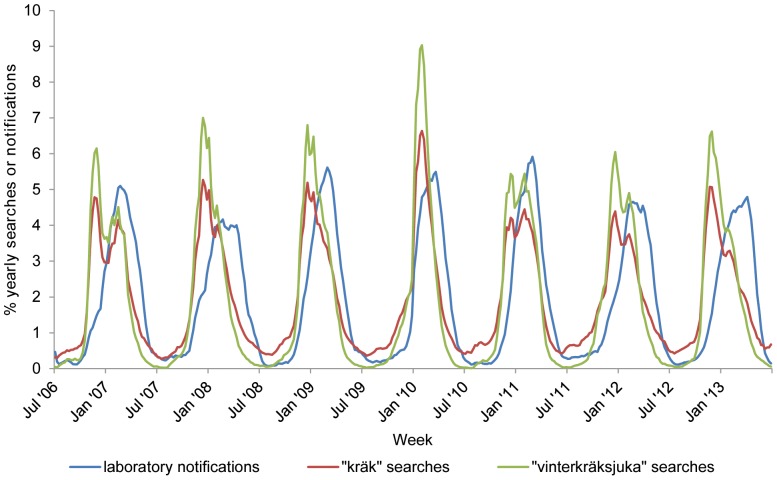
Weekly norovirus notifications and Websök searches, Sweden 2006–2013. Figure 1 represents for each week between week 27 2006 and week 26 2013, the norovirus notifications and online searches for the terms “kräk” (vomiting) and “vinterkräksjuka” (winter vomiting disease) expressed as weekly proportion of the yearly total after smoothing, standardisation and adjusting for trend. The blue line represents laboratory notifications, the red line represents online searches for “kräk” (vomiting) and the green line represents online searches for “vinterkräksjuka” (winter vomiting disease).

Between week 27 in 2006 and week 26 in 2013, laboratories reported 46,765 confirmed norovirus infections to SMI, and the terms “kräk” and “vinterkräksjuka” were searched 91,630 and 36,576 times respectively in vårdguiden.se. The total number of searches per year peaked in 2010 for both terms. After standardizing, adjusting for trend and smoothing, the number of laboratory notifications correlated with both the number of searches for “kräk” and “vinterkräksjuka”. (correlation coefficient  = 0.88 for “vinterkräksjuka”, 0.89 for “kräk” with a lag of 6 weeks for both search terms). Graphically, the trends for laboratory notifications and search terms were similar. However, the number of searches increased earlier in the year than laboratory notifications (figure 1). In addition, a double peak can be seen in the search term data (most clearly in the 2006–07, 2010–11 and 2011–12 seasons, Figure 1) but not in the laboratory notifications data.

### Timeliness

**Figure 2:**
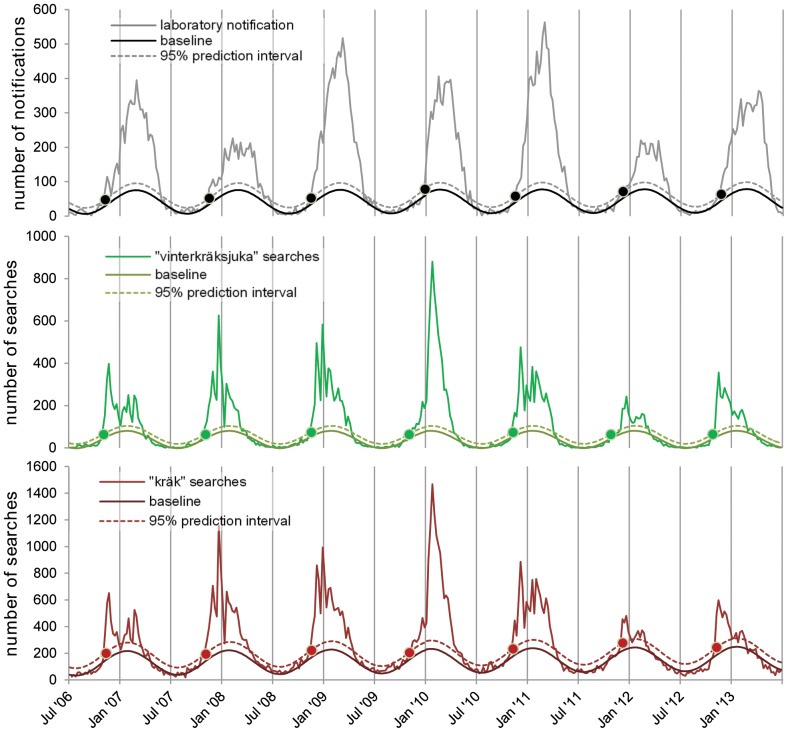
Number of weekly norovirus laboratory notifications and Websök searches, Sweden, 2006–2013. Dots represent the weeks the threshold for norovirus season onset is crossed. Figure 2 presents three separate graphs. The black graph represents laboratory notifications, the green graph represents online searches for the term “vinterkräksjuka” (winter vomiting disease) and the red graph represents online searches for the term “kräk” (vomiting). Each graph presents the number of notifications or searches per week between week 27 2006 and week 26 2013, along with a baseline and the 95% upper prediction interval line of the baseline. For each year, on each graph, the dots represents the week when the number of searches or notifications exceeded the 95% upper prediction interval line of the baseline, thus indicating when the onset of the norovirus season is detected in each case.

Compared with laboratory notifications, the number of searches for “kräk” and “vinterkräksjuka” exceeded the 99% upper prediction interval of the baseline activity an average of two weeks earlier (range 0–6, Table 1). When using the upper 95% prediction interval, the number of searches exceeded the threshold an average of two weeks earlier (range 0–8) for “kräk”, and three weeks earlier (range 0–8) for “vinterkräksjuka” (Figure 2). Of seven norovirus seasons, Websök detected the season onset earlier than laboratory data between 4 and 6 times, depending on the search term and threshold used (Table 1). Additionally, the number of searches for “kräk” exceeded the 95% threshold during low activity months for two seasons in a row (week 36 in 2009 and week 33 in 2010).

**Table 1:** Week number when activity crossed the epidemic threshold for norovirus laboratory notifications, and searches for “kräk” (vomiting) and “vinterkräksjuka” (winter vomiting disease) in Websök along with number of weeks gained for each search term compared with laboratory notifications, Sweden, 2006/2007 - -2012/2013.

		Week activity crossed epidemic threshold						Weeks gained			
		Laboratory		Kräk		Vinterkräksjuka		Kräk		Vinterkräksjuka	
Prediction interval		95%	99%	95%	99%	95%	99%	95%	99%	95%	99%
Season	2006–07	45	45	45	45	44	44	0	0	1	1
	2007–08	46	46	44	44	44	44	2	2	2	2
	2008–09	46	46	46	46	46	46	0	0	0	0
	2009–10	52	52	44	46	44	46	8	6	8	6
	2010–11	46	46	45	45	45	45	1	1	1	1
	2011–12	49	51	49	49	43	49	0	2	6	2
	2012–13	47	47	45	45	43	45	2	2	4	2

### Representativeness

Compared with the general Swedish population, Vårdguiden.se users were more likely to be female, university educated and aged 31–65 (p<0.001 for each, Table 2). Of the 17,323,214 visits to the website in 2012, 16,221,649 (94%) originated from Sweden. The geographical distribution of the visits differed from what the population distribution (p<0.001). Stockholm county accounted for 22% of the population but accounted for 46% of the domestic visits to the website.

**Table 2:** Sociodemographic characteristics of Vårdguiden.se users compared with the Swedish population, 2012.

		Vårdguiden users		Sweden		P value*
		Number (n = 3000†)	Proportion (%)	Number (n = 9 555 893)	Proportion (%)	
**Age**	0–10	0	0	1 117 576	12	<0.001
	10–17	90	3	810 545	8	
	18–30	570	19	1 643 022	17	
	31–50	1140	38	2 529 920	26	
	51–65	750	25	1 749 773	18	
	>65	450	15	1 705 057	18	
**Sex**	Male	690	23	4 765 905	50	<0.001
	Female	2280	76	4 789 988	50	
**Education**	Primary	240	8	1399764**	20	<0.001
	Secondary	1110	37	3120099**	45	
	University	1590	53	2374052**	34	

† Totals do not always add up to 3000 due to missing answers

*p value for the chi square goodness of fit test

** out of the 16–74 year old 2012 Swedish population, n = 6893915.

### Usefulness

Of 21 counties, 17 (81%) answered the survey. All were affected by norovirus outbreaks in the 2012–13 season, lasting up to 60 days and involving at least 2,664 patients and staff. During the 2012–13 norovirus season, 69% infection control teams closed wards, 69% had sick members of staff, 80% restricted staff to working on specific wards and 13% redirected patients to other hospitals (Table 3). Infection control teams reported a range of preventive and reactive measures when dealing with hospital norovirus outbreaks (Table 3). Regarding timing of infection control measures, 35% respondents began implementing infection control measures after receiving information that the winter vomiting season had started, 18% did so at a fixed date in October or November every year and 35% began once an outbreak was declared (Table 3). 12% teams received a warning to the beginning of the 2012–13 norovirus season and 56% actively searched for information regarding the beginning of the norovirus season (Table 3). 54% infection control teams considered web-based surveillance as trustworthy as laboratory data but 38% thought it was less trustworthy (Table 3). 52% of teams said they would trust web based surveillance information only as a complement to laboratory data (Table 3). 88% considered that a Websök based early warning would constitute useful information, 65% thought it would help them direct their infection control strategy and 31% stated it would help decrease the number or size of norovirus outbreaks in hospitals (Table 3).

**Table 3:** Impact of norovirus outbreaks in hospitals and perception of web-based surveillance data according to 17 Swedish infection control teams, 2012/13.

Impact of norovirus outbreaks on hospital	Closed wards	11/16	69
	Staff members off sick	11/16	69
	Staff restricted to specific wards	12/15	80
	Patients redirected to other hospitals	2/15	13
Beginning of infection control activities	After learning the winter vomiting season had started	6/17	35
	Fixed date every year (October or November)	3/17	18
	After the first outbreak was declared	6/17	35
	Other	2/17	12
Method infection control team became aware of beginning of norovirus season	Received a warning	2/16	12
	actively searched for the information	9/16	56
	Other	5/16	31
Preventive measures used for hospital norovirus outbreaks	Improved staff hand washing policy	6/9	67
	Improved patient handwashing policy	2/9	22
	Modified visiting rules	0/9	0
	Information in media (radio, TV, newspapers, internet	2/9	22
	Posters in hospitals	1/9	11
	Staff training	3/9	33
Reactive measures to hospital norovirus outbreaks	Improved staff hand washing policy	14/17	82
	Improved patient handwashing policy	1417	82
	Modified visiting rules	12/17	71
	Information in media (radio, TV, newspapers, internet	7/17	41
	Posters in hospitals	13/17	76
	Closed wards	10/17	59
	Isolation of infected patients	17/17	100
	Restricting patient movements between departments	15/17	88
	Protective equipment for staff	16/17	94
	Restricting staff to work on specific wards	9/17	53
	Daily communication between hospital and infection control team	12/17	71
	Deep cleaning of affected wards	1/17	6
General perception of web-based surveillance data	Web based surveillance data always trusted	2/17	12
	Web based surveillance data trusted if originates from official source	6/17	35
	Web based surveillance data trusted as complement to laboratory data only	9/17	53
Perception of web-based surveillance data compared with laboratory data	Web-based data and laboratory data as trustworthy	7/13	54
	Web-based data more trustworthy	1/13	8
	Web-based data less trustworthy	5/13	38
Perception of early warning from web-based surveillance data	Useful information	15/17	88
	Helps to direct infection control strategy	11/17	65
	Decreases the number or size of norovirus outbreaks in hospitals	5/16	31

## Discussion

We evaluated Websök, an internet-based surveillance system for norovirus, and found that it was a reliable, timely and useful tool to detect the onset of the norovirus season. Where search query data are readily accessible, it is a simple system to set up and practically free to run.

Trends in norovirus related online queries correlated with laboratory surveillance. This finding is consistent with results of investigations into other internet based surveillance systems, both for norovirus [22] and influenza [16]. The six week lag between laboratory data and Websök data in the cross-correlation analysis may be partly explained by a lag in laboratory reporting, partly by the delay between community circulation and circulation in hospitals, and partly by the fact that Websök is a high sensitivity, low specificity tool, potentially capturing events or episodes not attributable to norovirus and therefore occurring in a different timeframe. Since Websök is based on data from a health portal, it might be more adequate for outbreak detection that systems based on generic search engine data. However, it is not possible to equate one search in Websök with one norovirus case. Also, as searches in Websök are anonymous, it is not possible to differentiate for example between ten individuals searching information on winter vomiting disease and one individual searching for information ten times at different occasions. Likewise, Websök cannot differentiate between individuals who search because of their symptoms and those who are well and search out of general interest. Although the detection of the season onset was not influenced by other pathogens overall, the double peak seen in several seasons in the Websök data, but not in the laboratory data, could be caused by distinct pathogens, a phenomenon consistent with the literature [4]. For these reasons, interpretation of Websök data should be restricted to overall trends and detection of the season onset and cannot be extended to severity or magnitude. This restriction is further warranted by the reported over-estimation of outbreak magnitude when using search engine data [18], [19]. In light of these limitations, Websök should be seen as a complement, rather than an alternative, to laboratory surveillance.

Compared with laboratory surveillance, Websök detected the onset of the winter vomiting season two to three weeks earlier on average, depending on keyword and prediction interval used. This finding was consistent with studies from other countries [7], [22]. When evaluating different search terms, our results suggested that compared with a generic search term such as “kräk” (vomiting), a more specific search word such as “vinterkräksjuka” (winter vomiting disease) provided better results, for three reasons. First, “kräk”, as a generic term, is more sensitive and generated alerts during the low activity months which were not confirmed by laboratory data. Second, “vinterkräksjuka” achieved early detection of the norovirus season onset for more seasons than did “kräk”. Third, when we used the 95% upper prediction interval as the epidemic threshold, the term “vinterkräksjuka” detected the onset of the winter vomiting season earlier than with a higher prediction interval. However no combination of search term and epidemic threshold gave an early warning for all investigated seasons. When the delay between laboratory surveillance and Websök was particularly long, such as in the season 2009–10, the laboratory detection surveillance occurred later while the Websök detection did not occur earlier. In 2009–10 in France, the winter onset of the acute gastrointestinal illness season was delayed by 5 weeks [28]. In Sweden, it was unclear whether the discrepancy in that year is caused by a false positive detection in the search term data, a late detection using laboratory data for an unknown reason, or a longer lag between community circulation and hospital circulation.

An early signal per se is not an end in itself. The detection signal should reflect the epidemiology of the virus, and be based on plausible data. Too early a signal, disconnected from the norovirus season would have little public health value. Websök purports to reflect circulation of the virus in the community, which on average precedes detection by laboratory data, reflecting hospital circulation, by 2–3 weeks. This signal is close enough to the onset of nosocomial outbreaks to prompt infection control teams to act. Search engine data surveillance systems, such as Websök, have high sensitivity and low specificity by nature, with an inherent risk of false or artificially early signals. Websök mitigates this risk and increases its specificity by relying on a local health portal data and by using a search term, which, in Sweden, is strongly associated with norovirus.

Websök's users were not representative of the Swedish population. However, as our objective was to detect the nation-wide onset of the norovirus season rather than to precisely estimate the incidence of disease, this lack of representativeness is unlikely to bias the overall detection of the season onset. One consequence of Websök's lack of representativeness is that the early signal may not apply to all Swedish regions since season onset is influenced by climate, which may vary in different parts of Sweden at any given time. In December 2005 for example, southern Sweden experienced increasing norovirus activity, while the rest of the country reported relatively low activity [24]. In our evaluation, the detection of the norovirus season onset was likely biased towards Stockholm since users from that area were over-represented.

For a surveillance system to be useful, it needs to lead to public health action. The perceived usefulness by the intended end-users is of particular interest. Our survey provided important information on the public health usefulness of early detection and the current preparedness activities and attitudes. Most infection control teams believed that an early warning to the onset of the winter vomiting disease season would help them plan their infection control strategies. However, infection control teams perceived web-based data as less reliable than laboratory data. Therefore, these teams are likely to take both the Websök signal, earlier and less specific, and the laboratory-data signal, later and more specific, into consideration when planning infection control strategies. As of 2012, less than half of the infection control teams in Sweden who responded to our survey used surveillance-based evidence to plan their norovirus infection control activities. The majority started their infection control activities either at a fixed point in time or once outbreaks had occurred. If these teams were to use Websök's early warning, they would be able to implement earlier infection control measures which could reduce the number and size of outbreaks.

## Conclusions and Recommendations

Websök provides surveillance data that detect the onset of the norovirus season as reliably as laboratory data, but earlier. In our survey, infection control teams viewed this early signal as useful for planning infection control measures, although they perceived web based surveillance data as less reliable than laboratory based data. Since this evaluation we have integrated Websök in routine norovirus surveillance, along with laboratory surveillance. We have also improved the collaboration between the national centre for communicable disease control and local infection control teams regarding norovirus season detection. Issue of regular newsletters and educational events could potentially increase the local infection control teams' confidence in Websök and ensure its use. Since 2013, we send email alerts to all infection control teams in Sweden when the number of searches for “vinterkräksjuka” first exceeds the upper limit of the 95% prediction interval of the baseline search activity for the season. The use of internet systems such as Websök may be replicated at low cost in countries where internet access is widespread. Based on our evaluation, we recommend (i) the use of a Websök-like surveillance system as a complement to laboratory surveillance, in countries where data for search engines is available and where norovirus is a public health concern (ii) the use of search engine data for the detection of the norovirus season using a local health-focused search engine if possible (iii) a close collaboration with local infection control units to ensure the data is understood, trusted and used optimally for early implementation of infection control measures.
